# Nationwide survey to evaluate the decision-making process in euthanasia requests in Belgium: do specifically trained 2nd physicians improve quality of consultation?

**DOI:** 10.1186/1472-6963-14-307

**Published:** 2014-07-16

**Authors:** Joachim Cohen, Yanna Van Wesemael, Tinne Smets, Johan Bilsen, Bregje Onwuteaka-Philipsen, Wim Distelmans, Luc Deliens

**Affiliations:** 1 End-of Life Care Research Group, Vrije Universiteit Brussel (VUB) & Ghent University, Brussels, Belgium; 2 Department of Public Health, Vrije Universiteit Brussel (VUB), Brussels, Belgium; 3 VU University Medical Center, Department of Public and Occupational Health, EMGO Institute for Health and Care Research, Expertise Center for Palliative Care, Amsterdam, The Netherlands; 4 Universitair Ziekenhuis Brussel, Supportive & Palliative Care, Brussels, Belgium

**Keywords:** Euthanasia, Consultation, Referral practice, Terminal care

## Abstract

**Background:**

Following the 2002 enactment of the Belgian law on euthanasia, which requires the consultation of an independent second physician before proceeding with euthanasia, the Life End Information Forum (LEIF) was founded which provides specifically trained physicians who can act as mandatory consultants in euthanasia requests. This study assesses quality of consultations in Flanders and Brussels and compares these between LEIF and non-LEIF consultants.

**Methods:**

A questionnaire was sent in 2009 to a random sample of 3,006 physicians in Belgium from specialties likely involved in the care of dying patients. Several questions about the last euthanasia request of one of their patients were asked. As LEIF serves the Flemish speaking community (i.e. region of Flanders and the bilingual Brussels Capital Region) and no similar counterpart is present in Wallonia, analyses were limited to Flemish speaking physicians in Flanders and Brussels.

**Results:**

Response was 34%. Of the 244 physicians who indicated having received a euthanasia request seventy percent consulted a second physician in their last request; in 30% this was with a LEIF physician. Compared to non-LEIF physicians, LEIF physicians were more often not a colleague (69% vs 42%) and not a co-attending physician (89% vs 66%). They tended to more often discuss the request with the attending physician (100% vs 95%) and with the family (76% vs 69%), and also more frequently helped the attending physician with performing euthanasia (44% vs 24%). No significant differences were found in the extent to which they talked to the patient (96% vs 93%) and examined the patient file (94% vs 97%).

**Conclusion:**

In cases of explicit euthanasia requests in Belgium, the consultation procedure of another physician by the attending physician is not optimal and can be improved. Training and putting at disposal consultants through forums such as LEIF seems able to improve this situation. Adding stipulations in the law about the necessary competencies and tasks of consulting physicians may additionally incite improvement. Irrespective of whether euthanasia is a legal practice within a country, similar services may prove useful to also improve quality of consultations in various other difficult end-of-life decision-making situations.

## Background

Euthanasia, i.e. the intentional ending of life by a physician at a person’s explicit request, has been legal in Belgium under strict conditions since 2002 [1,2]. The person must be in a medically hopeless situation of persistent and unbearable physical or psychological suffering as a consequence of a serious and incurable medical condition, which cannot be alleviated otherwise. His or her request must be voluntary, well-considered and repeated. One of the procedural conditions to be followed before considering euthanasia is that the attending physician has to consult a second physician, the consultant, who must be independent from both the attending physician and the patient. This consultant has to read the medical file, examine the patient and ascertain that the patient’s suffering is unbearable and the request is voluntary, well-considered and repeated. He also has to write a report about his judgement. These are due care criteria to guarantee the safe practice of euthanasia by means of a control mechanism beforehand. If the attending physician and/or the consultant do not anticipate death of the person requesting euthanasia in the near future (e.g. in case of a quadriplegic person, or a person suffering from MS), a third independent physician who is a specialist in the disease or a psychiatrist, must be consulted and must perform the same tasks as the other consultant. After euthanasia has been performed, the attending physician must report it to the Federal Control and Evaluation Commission on Euthanasia using an available standard form, which serves as an a posteriori control mechanism. In 2009, for instance, a total of 822 euthanasia cases were officially reported to this commission [3]. A large scale population-based representative study from 2007 estimated the total number of possible euthanasia cases to be almost twice the number of reported cases (although unreported cases were often not considered to be euthanasia by the physician) [4]. Concerns have been raised about the actual adherence to the legal criteria of both the a priori consultation and a posteriori reporting [5].

While the legislator regulated the consultation procedure in euthanasia requests it did not accommodate this procedure, for instance by providing adequate training for physicians to assess the due care criteria and other complex circumstances that would make a euthanasia legally acceptable. In Flanders, the Dutch speaking part of Belgium, a special service called Life End Information Forum (LEIF) was then established as a non-governmental initiative to provide information and training for health care professionals in end-of-life care matters such as euthanasia, since many did not have any experience in these matters [6-9]. In 2003, the forum started organizing specific training for physicians to obtain the necessary skills and knowledge to act as independent consultants in euthanasia requests [6]. The idea was that if a second physician was needed to appraise a request, that physician better had the necessary qualifications regarding both euthanasia and palliative care. As palliative care receives insufficient attention in the regular medical curriculum, and basic palliative care skills and knowledge were deemed necessary to evaluate options in a euthanasia request the training also paid attention to palliative care apart from euthanasia [10-14]. On completing the training, these physicians become LEIF physicians. Attending physicians who receive a euthanasia request and who want to consult with an independent physician can call a central telephone number and a LEIF physician within their region is then assigned to them, or they can contact a LEIF physician directly. The Belgian euthanasia law does not specify, of course, that the consultant has to be a LEIF physician. LEIF is a Dutch speaking initiative and hence serves the Flemish speaking community, which lives in the regions of Flanders and the official bilingual Brussels Capital Region. No initiative of similar magnitude and organisation like LEIF is present in Wallonia.

Previously published data on how euthanasia requests are granted or not in Belgium have demonstrated that the advice of a second physician (the consultant) plays a key role in a euthanasia request being granted [15]. In this study, we want to examine how often the consultant is a LEIF physician and whether consultation with a LEIF physician improves the quality of the consultation, by answering the following research questions:

1) How often is a LEIF physician consulted in euthanasia requests in Flanders and Brussels - the area covered by the LEIF physicians- and what are characteristics of the attending physician associated with consulting a LEIF physician instead of a non-LEIF physician?

2) To what extent are the legal requirements met in consultations with a second physician and is there a difference between those with LEIF and non-LEIF physicians?

3) Is there a difference in the consultations between LEIF and non-LEIF physicians in relation to additional non-mandatory consultation features (e.g. conversation with the family) and the outcome of the consultation (e.g. judgement about whether due care criteria were met and whether euthanasia was performed)?

## Method

### Study design

In March 2009 we sent a questionnaire to a random sample of 3,006 Belgian physicians by mail. The sampling frame was a weekly updated commercial register based on the register of the National Institute for Health and Disability Insurance (NIHDI). This commercial register was chosen because the prevailing privacy law made official registers from the NIHDI unavailable to researchers and because the quality and completeness of the register was judged as high based on a comparison with aggregate distributions from the NIHDI data. From this sampling frame we only selected registered medical practitioners who worked in Belgium, had graduated in their speciality at least 12 months before the sample was drawn, and were likely to be involved in the care of dying patients. The latter selection was done based on specialty. Those specialities for which little or no experience in the care for the dying could be expected were excluded. The following specialities were included: general practice, anaesthesiology, gynaecology, internal medicine, neurology, pulmonary medicine, gastroenterology, neuropsychiatry, psychiatry, cardiology, radiotherapy, and surgery. The random sample was stratified for province and speciality and represents a sampling fraction of 9.2%.

The researchers sent a questionnaire with a unique serial number to each physician in the sample. The physicians were instructed in a covering letter to send the questionnaire to an independent lawyer, guaranteeing complete anonymity while allowing for the sending of up to three reminders [16]. The anonymity procedure and study protocol were approved by the Ethical Review Board of the University Hospital of the Vrije Universiteit Brussel.

To assess non-response bias, non-responders were sent a one-page form asking them for their reasons for not participating and requesting them to fill in two key questions from the original questionnaire, one about their attitude towards euthanasia, and another about their experience with euthanasia requests [17].

### Questionnaire

The pre-structured, eight-page questionnaire with mainly closed-end questions was partly based on one previously used in the Netherlands [18]. The questions were adapted to make them appropriate for the Belgian legal context and culture. Concerning their most recent euthanasia request, physicians were asked to answer questions on patient and request characteristics, consultation with a second physician, activities of the second physician and outcome of the request. Questions on quality criteria in accordance with the Belgian due care criteria for consultation were included (see List of quality criteria section) [19]. In the questionnaire, euthanasia was defined as ‘the intentional ending of the patient’s life at his/her explicit request by the physician’; this definition corresponds to the legal definition of euthanasia in Belgium.

#### **List of quality criteria as outlined by the Dutch consultation protocol**

The consultant

– was not a colleague of the attending physician

– was not a co-attending physician

– did not know the patient

– talked to/examined the patient

– considered the request*

– talked about possible alternatives†

– made a written report

*This criterion was not questioned in the Belgian survey because it is too vague and not a task stipulated in de Belgian euthanasia law.

†This criterion was not questioned in the Belgian survey because it is not a requirement for the second physician in the Belgian law on euthanasia.

### Statistical analysis

For all analyses we selected only the responses from Dutch-speaking physicians from Flanders and Brussels, because LEIF offers its services and trainings in Dutch and provides its services in Brussels and Flanders. Fisher exact tests were performed to compare for LEIF and non-LEIF physicians’ independence and activities. P-values that were less than 0.05 were considered statistically significant. The analyses were performed using SPSS 19.0.

## Results

### Response rate and response bias

Of the 3,006 questionnaires sent, 222 physicians were unreachable, deceased or no longer in practice. From the non response survey another 57 were identified as no longer practising or not having received the questionnaire. As such, there were 2,726 eligible physicians from whom 914 questionnaires (34%) were returned. Small but significant differences between the responders of the survey and responders of the non-response survey were found for attitude toward euthanasia (90.4% vs 87.4% agreed on the statement concerning attitude toward euthanasia). No differences between these groups were found concerning having ever received a request.

### Physicians receiving euthanasia requests and consulting a second physician

Two hundred and forty-four Dutch-speaking physicians from Flanders and Brussels had received a euthanasia request since 2002 and described the most recent request they had received. Seventy per cent of these respondents had consulted with a second physician about this request (Table 1); for the cases where euthanasia was actually performed (N = 123) consultation had taken place in 91.9% (not in table). In 51 (30.0%) of the consultations, the consultant was a LEIF physician. General practitioners more often than specialists consulted with a LEIF physician. Physicians between 36 and 50 years old had significantly more often consulted a second physician than had their younger and older colleagues but this was less often a LEIF consultant.

**Table 1:** **Number of consultations with a second physician** (**LEIF and not LEIF**) **since the euthanasia law**, **according to physician**’**s characteristics** (**n** = **244**)

	Number of physicians who consulted with a second physician after receiving request in FL and BXL (and % of total physicians in FL and BXL)* Total N = 170 (69.7%)	**p-value**^**†**^	**LEIF physician N = 51 (30.0%)**	**Not LEIF physician or not known N = 119 (70.0%)**	**p-value LEIF vs not LEIF**^**‡**^
Specialty					
General practitioners	118 (69.4)	0.764	42 (35.6)	76 (64.4)	**0.010**
Specialist	51 (30.2)		9 (15.7)	43 (84.3)	
Physician’s age					
Younger than 36	17 (10.1)	0.656	5 (29.4)	12 (70.6)	1.000
36-50 years	74 (44.0)	0.022	14 (18.9)	60 (81.1)	0.017
51-60 years	58 (34.5)	0.149	22 (37.9)	36 (62.1)	0.153
Older than 60	19 (11.3)	0.526	9 (47.4)	10 (52.6)	0.114
Number of terminal patients cared for in 1 year					
0 patients	12 (7.2)	0.202	6 (50.0)	6 (50.0)	0.102
1 to 10 patients	114 (74.5)	0.255	35 (30.7)	79 (69.3)	1.000
more than 10 patients	27 (17.6)	0.850	6 (22.2)	21 (77.8)	0.360
Religiosity					
Not religious	57 (33.5)	0.176	19 (33.3)	38 (66.7)	0.595
Religious	113 (66.5)		32 (28.3)	81 (71.7)	
Training in palliative care or member of palliative team					
Yes	115 (67.6)	0.243	38 (33.0)	77 (67.0)	0.283
No	55 (32.4)		13 (23.6)	42 (76.4)	

*1 to 17 missing cases. *FL* = Flanders, *BXL* = Brussels.

^†^Comparison with physicians who did not consult. Fisher exact test for statistically significant differences between categories vs all other categories within the variable. Significant differences in bold.

^‡^Fisher exact test for statistically significant differences between categories vs all other categories within the variable.

### Quality of consultation

Table 2 lists the extent to which the Dutch criteria for a good consultation were met by all physicians and by LEIF physicians. For all physicians, the consultant was not a direct colleague (i.e. same working environment) of the attending physician in 41.8% of cases and in two thirds of cases the consultant was not a co-attending physician of and did not know the patient. These criteria of independence in relation to both the attending physician and the patient were met significantly more often when the consultant was a LEIF physician as compared with a non-LEIF physician (respectively 68.6%, 88.7%, and 86.3% compared to 30.3%, 53.8%, and 49.2%). No significant differences were found in the extent to which they talked to the patient (96% vs 93%), examined the patient file (94% vs 97%), or made a written report about their judgement regarding the euthanasia request (73% vs 67%).

**Table 2:** Extent to which criteria for quality of consultation are met according to whether the second physician is a LEIF physician or not

	All* N = 170	**LEIF physician (N = 51)**^**†**^	**Not LEIF physician (N = 119)**^**‡**^	**p-value LEIF vs not-LEIF**
The second physician				
Was not a colleague of the attending physician	71 (41.8)	35 (68.6)	36 (30.3)	< 0.0001
Was not a co-attending physician	111 (65.7)	47 (88.7)	64 (53.8)	< 0.0001
Did not know the patient	102 (60.4)	44 (86.3)	58 (49.2)	< 0.0001
Examined the patient file	161 (95.8)	48 (94.1)	113 (96.6)	0.435
Talked to/examined the patient	155 (93.9)	48 (96.0)	107 (93.0)	0.725
Made a written report	106 (62.8)	35 (72.9)	71 (67.0)	0.456

*1 to 16 missing cases.

^†^1 to 3 missing cases.

^‡^1 to 13 missing cases.

All the LEIF physicians had discussed the request with the attending physician compared with 94.9% of the non-LEIF physicians (difference not statistically significant; Table 3). LEIF physicians tended to less often discuss the request with another attending physician or with a third physician than had non-LEIF physicians, although the difference was not statistically significant. LEIF physicians had helped the attending physician with performing the euthanasia more often than had non-LEIF physicians and tended to more often help with filling out the reporting form.

**Table 3:** Additional tasks performed by the second physician according to whether the second physician is a LEIF physician or not

	All (N = 170)*	**LEIF physician (N = 51)**^**†**^	**Not LEIF physician or not known (N = 119)**^**‡**^	**p-value LEIF vs non-LEIF**
The consultant				
Had a discussion with the attending physician	161 (96.4)	50 (100.0)	111 (94.9)	0.180
Had a conversation with the family	114 (71.3)	37 (75.5)	77 (69.4)	0.456
Had a conversation with the caring team	80 (51.0)	23 (46.9)	57 (52.8)	0.606
Had a conversation with another attending physician	55 (35.9)	10 (20.8)	45 (42.9)	0.011
Asked for additional advice from a third physician	29 (18.7)	5 (10.4)	24 (22.4)	0.117
Was present when euthanasia was performed	45 (30.4)	17 (37.0)	28 (27.5)	0.253
Helped with performing euthanasia	46 (30.1)	21 (43.8)	25 (23.8)	0.022
Helped with filling out the registration form	54 (36.0)	22 (45.8)	32 (31.4)	0.102

*2 to 22 missing cases.

^†^1 to 4 missing cases.

^‡^1 to 16 missing cases.

### Advice of the consultant and outcome of the request

The consultant gave a positive advice (i.e. concluded that the conditions for euthanasia were met) in 80.5% (N = 136) of all requests (Table 4).

**Table 4:** Advice of consultant according to whether the second physician is a LEIF physician or not

	All (N = 170)*	**LEIF physician (N = 51)**	**Not LEIF physician or not known (N = 119)**^**†**^	**p-value LEIF vs non-LEIF**
Consultant concluded that conditions for euthanasia were met	136 (80.5)	46 (90.2)	90 (76.3)	0.037
Extent to which the judgment of the consultant played a part				
To a great extent	57 (33.5)	20 (39.2)	37 (31.1)	0.478
To some extent	45 (26.5)	9 (17.6)	36 (30.3)	0.124
Hardly	26 (15.3)	11 (21.6)	15 (12.6)	0.157
Not at all	39 (22.9)	11 (21.6)	28 (23.5)	0.843

*3 missing cases.

^†^1 to 3 missing cases.

Overall, LEIF physicians significantly more often gave a positive advice (i.e. concluded that the conditions for euthanasia were met) compared with non-LEIF physicians (90.2% vs 76.3%). When asked to what extent the advice of the consultant had influenced their final decision, 60% of respondents indicated that it had to some or to a great extent (56.8% LEIF and 61.4% non-LEIF).

Sixty-eight percent (N = 113) of the requests described in Flanders and Brussels resulted in euthanasia. Euthanasia was more often performed when a LEIF physician as opposed to a non-LEIF physician had been consulted but this difference was not significant (76.0% vs 64.1%, p = 0.151, not in table). Also, euthanasia was more often reported in case the consultant was a LEIF physician as compared to a non-LEIF physician but this difference was not significant either (86.5% vs 77.3%, p = 0.317, not in table).

## Discussion

This study is the first to examine the quality of consultation between attending physicians and consultants in euthanasia requests in Belgium. We found that in 70% of the euthanasia requests described the attending physician had consulted with a second physician. Of the (Dutch) criteria for good practice, independence of the consultant, either from the physician or the patients, is the one most often unmet. Life End Information Forum (LEIF) physicians, who had undergone training as consultants in euthanasia requests, were significantly more often independent from the attending physician and from the patient compared with those who had not received the LEIF training.

An important strength of this study is that we used a large sample of physicians from diverse specialties to ask questions about a very sensitive subject. To this end, we used a rigorous sampling and mailing procedure. The questionnaire was comprehensively tested. There are however also some limitations. First, the low response percentage makes it difficult to generalize the results to all physicians in Flanders and Brussels, although analyses of the non-response survey indicate that the sample of responders was comparable to the sample of non-responders regarding having received a euthanasia request. As the survey is retrospective, there may be recall bias, especially for requests from more than a year earlier. Furthermore, the information provided in this survey on the activities of the consultants stems only from the attending physician. An additional limitation is that, with the absence of an initiative of similar magnitude and organisation present in Wallonia or for French speaking Belgium, the analyses had to be limited to Flemish speaking physicians in Flanders and Brussels. The presence of an initiative for Flemish speaking physicians but absence of one for French speaking physicians may reflect the fact that Flanders shares the language of and is culturally closer to the Netherlands. In addition, a previous study also indicated more positive attitudes among Flemish physicians towards the due care criteria of the euthanasia law and a larger willingness to comply with these criteria [20]. As regional differences in the euthanasia practice likely also reflect cultural differences [20] a comparison of LEIF physicians versus non-LEIF physicians for the whole of Belgium (with all French speaking physicians thus being in the non-LEIF group) would have been contaminated by these differences. Hence, although it is likely that a service like LEIF would have similar positive effects in French speaking Belgium, our results cannot just be generalized to French speaking Belgium.

We found that 70% of the physicians who had received a euthanasia request had consulted with a mandatory second physician and for the cases where euthanasia was eventually performed this was 92%. A previous study in the Netherlands found consultation to take place in 87% of requests reported by GPs in the period 2000–2002 and in 97% of cases where euthanasia was performed [21]. Compared with the Netherlands, consultation in Flanders is thus rather low, especially when taking into account that the percentages in the Netherlands date from a period before 2002, when euthanasia was tolerated but not yet legalized in this country. That attending physicians do not consult a second physician in 30% of euthanasia requests can partly be attributed to the fact that in some cases they have already decided not to grant the request and consider they do not need a colleague to confirm their decision. It is open to discussion whether physicians need to consult with colleague physicians about every euthanasia request even if they feel it is not serious enough or feel reluctant to comply with it. The 8% of performed cases of euthanasia where no consultation took place are, however, problematic and do not comply with the legal due care criteria. Not consulting in these cases can possibly be attributed to a lack of knowledge of the procedure or to the reluctance of attending physicians to be scrutinized by a colleague [22].

Consultants in Flanders and Brussels were found not always to have examined or talked to the patient and a written report was not made in over one third of consultations. As these are prescribed in the euthanasia law as tasks of the consultant, it is surprising that these are not always met. It may not be possible to talk to all patients, especially if they have lost competency in decision-making (a situation that can also qualify for euthanasia if there is a previous written request or euthanasia declaration); however, our question also asked about an examination of the patient and it could be expected that at least this would have been done in patients who lost competency. The number of cases where neither were done is, however, very low. The cases without a legally required written report is a more frequent problem and may be due to the lack of knowledge about this legal criterion and a lack of legal control mechanisms to ascertain that a written report is made. An additional potential problem is that independence in relation to the physician and patient seemed often not guaranteed. In one third of cases the consultant was a co-attending physician of the patient. While the law does not describe precisely what is meant by independence [1], being a co-attending physician seems incongruent with the intention of the law that the consultant is able to give independent advice without influence from the attending physician or the patient. The fact that attending physicians do sometimes not seek an independent consultant could indicate that they consider the consultation merely a formality.

Our study indicated that involving consultants who followed a special training instead of those who did not could contribute to various aspects of the quality of consultation. The most important contribution seems to be in terms of the legally required independence: the LEIF physicians in our study were more often independent than non-LEIF physicians from the attending physician and the patient (i.e. they were more often unacquainted with them). The contact procedure through the central telephone number of LEIF, which is intended to assign a random LEIF physician (preferably from the region) to the attending physician, seems to create a better guarantee of independence as opposed to simply consulting a colleague from the same hospital or practice. However, this service appears to be called upon in particular by general practitioners, and much less often by specialists in hospitals who often call on their direct colleagues, which may be more practical and private but jeopardizes independence. Specialists therefore might especially benefit from a service such as LEIF in order to comply fully with this aspect of the law.

Although differences were not statistically significant, LEIF physicians also tended to more often discuss the request with the attending physician and the family than non-LEIF physicians. While these are not strict due care requirements stated in the law or the Dutch consultation protocol [19] they can also be seen as aspects determining the quality of a consultation, contributing to a more careful and qualitative consultation practice.

Involving specifically trained consultants can thus benefit euthanasia consultations in a number of aspects. A number of found differences between LEIF and non-LEIF consultants are perhaps not necessarily indicative of differences in the quality of consultation but nevertheless suggest relevant differences in consultation practice that merit further discussion. While the fact that LEIF-physicians less frequently discussed euthanasia requests with other attending physicians (i.e. colleague-attending physicians) is a result of the fact that LEIF physicians are more often consulted by GPs rather than by hospital specialists, the most striking difference with non-LEIF consultations is that LEIF consultations significantly more often led to a positive advice on the euthanasia request (i.e. a judgement that the conditions for euthanasia have been met) and significantly more often involved help with performing euthanasia. It may be that these differences reflect a more positive attitude towards euthanasia in general in LEIF physicians. Not being fundamentally against euthanasia is a prerequisite to being admitted to the LEIF training programme [6]. It has to be acknowledged that underlying attitudinal aspects may result in different outcomes of a consultation, which is inherent to the fact that assessing the nature and validity of a euthanasia request does not merely involve checking off objective criteria but also involves a great deal of subjective and compassionate judgement. While some may find in this finding a proof for too high a compliance with euthanasia requests in LEIF physicians, others will argue that the higher reluctance of non-LEIF consultants to give a positive advice about a euthanasia request is due to their larger uncertainty about the due care criteria and the procedure since they lack the specific training and similar experience.

Similarly some will argue that helping with the performance of euthanasia is not and cannot be the responsibility of a consultant whereas others will see it as a pedagogic assistance for attending physicians. The function of role models in medical education, particularly in end-of-life care, and their influence on ethical decision-making has been described in other studies [23-27] and previous research indicated that LEIF consultants help with euthanasia for medico-technical reasons, because they consider it important that a more experienced colleague can show the attending physician how to perform a delicate act they are not prepared for through the medical curriculum [7].

The model of LEIF, as a training and consultation service, is strongly based and inspired on the model of SCEN (Support and Consultation for Euthanasia in the Netherlands) in the Netherlands [6], which was already developed prior to the euthanasia legalisation in 2002 in the Netherlands [28]. The fact that SCEN is more regulated, organised on a larger scale, more endorsed by government, and puts more focus on formal aspects of the consultation (e.g. the written consultation report) [6], but also the more specific stipulations in the Dutch euthanasia law about the activities that are required by a consultant in a euthanasia request may be explanations for the larger compliance with legal requirements in SCEN physicians and in the Netherlands in general [29].

The euthanasia law in Belgium has recently (and only after our study was conducted) been adapted to include competent minor patients [30]. Although the practice of euthanasia in minor patients will likely continue to be a very rare and marginal practice (a previous study including all deaths of minor persons in Flanders during one and a half year identified no case of euthanasia [31]), the specific difficulties and challenges a careful euthanasia practice in this population poses will require LEIF to pay specific attention to euthanasia requests in minor patients in its trainings.

## Conclusion

In cases of explicit euthanasia requests in Belgium, the consultation of an independent physician by the attending physician is not optimal and can be improved. Firstly, the proportion of consultations should be higher and secondly, there should be the required independence between the consultant and attending physician. The drafting of a written report about the judgement regarding the eligibility of the euthanasia request also needs to be more standardly performed by consultants. Although other legally prescribed consultation activities, such as a conversation or at least an examination of the patient, are almost always abided by, the norm should be 100%.

As we have demonstrated in this study, a service like the Life End Information Forum (LEIF) can contribute in some respects, by providing independent consultants but also by educating physicians on the consultation procedure in euthanasia requests. Adding stipulations in the law about the necessary competencies and tasks of consulting physicians may additionally incite improvement. Based on our findings a similar initiative to LEIF for French speaking physicians in Belgium seems warranted. While our findings have particular relevance to Belgium and other countries or regions having or considering legislation on euthanasia or assisted suicide, we believe that our findings have a wider relevance in pointing out how a consultation service and specific consultation training for various difficult end-of-life situations can contribute to end-of-life decision-making.

## Competing interests

Non-financial competing interests: Wim Distelmans is chair of the LEIF forum. He collaborated to the manuscript by providing all necessary information about the LEIF physicians and insights about possible explanations for the findings. However, he did not have any influence on the choice of data being presented and he committed a priori to not influence the interpretations the other authors chose to give to the findings.

## Authors’ contributions

JC, YVW, TS, JB, and LD designed the study. JC and LD obtained funding. JC, YVW, and TS were responsible for data acquisition and the data analyses. All authors contributed to the interpretation of data. JC and YVW drafted the manuscript and all authors critically revised the manuscript for intellectual content. All authors read and approved the final manuscript.

## Pre-publication history

The pre-publication history for this paper can be accessed here:

http://www.biomedcentral.com/1472-6963/14/307/prepub
